# Pathogenicity and Genetic Characterization of Vietnamese Classical Swine Fever Virus: 2014–2018

**DOI:** 10.3390/pathogens9030169

**Published:** 2020-02-28

**Authors:** SeEun Choe, Van Phan Le, Jihye Shin, Jae-Hoon Kim, Ki-Sun Kim, Sok Song, Ra Mi Cha, Gyu-Nam Park, Thi Lan Nguyen, Bang-Hun Hyun, Bong-Kyun Park, Dong-Jun An

**Affiliations:** 1 Viral Disease Division, Animal and Plant Quarantine Agency, Gimcheon, Gyeongbuk 39660, Korea; ivvi59@korea.kr (S.C.); shinjibong227@gmail.com (J.S.); kisunkim@korea.kr (K.-S.K.); ssoboro@naver.com (S.S.); rami.cha01@korea.kr (R.M.C.); changep0418@gmail.com (G.-N.P.); hyunbh@korea.kr (B.-H.H.); parkx026@korea.kr (B.-K.P.); 2 College of Veterinary Medicine, Vietnam National University of Agriculture, Hanoi 100000, Vietnam; letranphan@gmail.com (V.P.L.); nguyenlan@vnua.edu.vn (T.L.N.); 3 College of Veterinary Medicine and Veterinary Medical Research Institute, Jeju National University, Jeju 63243, Korea; kimjhoon@jejunu.ac.kr; 4 College of Veterinary Medicine, Seoul University, Gwanak-ro, Gwanak-gu, Seoul 08826, Korea

**Keywords:** CSFV, genotype, virulence, E2 gene, phylogenetic tree

## Abstract

Here, we examined the pathogenicity and genetic differences between classical swine fever viruses (CSFV) isolated on pig farms in North Vietnam from 2014–2018. Twenty CSFV strains from 16 pig farms were classified as genotype 2 (sub-genotypes 2.1b, 2.1c, and 2.2). The main sub-genotype, 2.1c, was classified phylogenetically as belonging to the same cluster as viruses isolated from the Guangdong region in South China. Strain HY58 (sub-genotype 2.1c), isolated from pigs in Vietnam, caused higher mortality (60%) than the Vietnamese ND20 strain (sub-genotype 2.2). The Vietnamese strain of sub-genotype 2.1b was estimated to have moderate virulence; indeed, genetic analysis revealed that it belongs to the same cluster as Korean CSFV sub-genotype 2.1b. Most CSFVs circulating in North Vietnam belong to sub-genotype 2.1c. Geographical proximity means that this genotype might continue to circulate in both North Vietnam and Southern China (Guangdong, Guangxi, and Hunan).

## 1. Introduction

Classical swine fever virus (CSFV), which belongs to the genus *Pestivirus* within the family *Flaviviridae*, is an enveloped virus containing a single-stranded, positive-sense RNA genome of approximately 12.3 kb [1]. The virus causes a highly contagious disease in pigs, which results in significant economic losses to the pig industry both in Vietnam and worldwide. Depending on the strain, CSFV can cause an acute, subacute, chronic, or asymptomatic disease [2,3]. Highly virulent CSFV results in high morbidity and mortality, whereas low virulent CSFV may cause asymptomatic disease [2,3]. CSFV is categorized into three genotypes (1, 2, and 3), each comprising three to four sub-genotypes (1.1–1.4, 2.1–2.3, and 3.1–3.4) [4]. CSFV strains isolated in China during the 1990s clustered into sub-genotypes 1.1, 2.1, 2.2, and 2.3 [5]; however, the majority of CSFV strains belonged to either sub-genotype 2.1 (49.1%) or 2.2 (36.4%). In Vietnam, some pigs have been vaccinated against CSFV; however, a previous study suggested that antibody responses against the CSF vaccine was significantly reduced in Trypanosoma (T) evansi-infected pigs as compared to uninfected pigs. This immunosuppression might explain the accounts of poor protection of CSF-vaccinated pigs reported in T. evansi endemic areas of Vietnam [6]. Genetic analysis of the 5’ non-translated region (5’ NTR) of CSFVs isolated in the Mekong delta area of South Vietnam between 2001 and 2003 showed that the strains clustered into two sub-genotypes: the majority clustered into sub-genotype 2.1, with the remainder clustering into sub-genotype 2.2 [7]. In Thailand, a country neighboring Vietnam, all three sub-genotypes (2.1, 2.2, and 2.3) were isolated throughout the 1980s and early 1990s, but sub-genotype 2.2 was the major isolate from 1996 onwards [8]. CSFVs isolated in the northern provinces of Laos (bordering Vietnam) between 1997 and 1999 belonged to sub-genotype 2.1, whereas most of those isolated in the southern provinces of Laos belonged to subgroup 2.2; CSFVs isolated in central provinces of Laos belonged to sub-genotypes 2.1 and 2.2 [9]. The geographical position of Vietnam makes it particularly vulnerable to CSF outbreaks because pigs come into the country across the borders with Cambodia, Laos, and China. 

The objective of the present study was to examine the pathogenicity and phylogenetic genotypes of CSFVs circulating recently in northern Vietnam.

## 2. Results 

### 2.1. Genotype Analysis and Phylogenetic Tree

**Figure 1:**
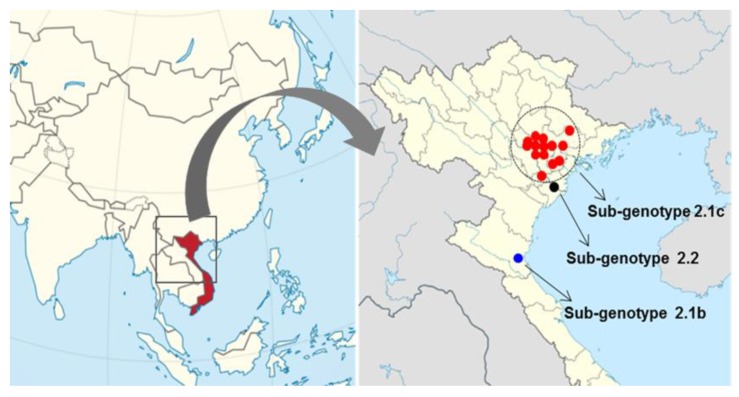
Map showing outbreaks of classical swine fever on pig farms in northern Vietnam. CSFV genotypes are denoted by the red circle (sub-genotype 2.1c), black circle (sub-genotype 2.2), and blue circle (sub-genotype 2.1b).

**Figure 2:**
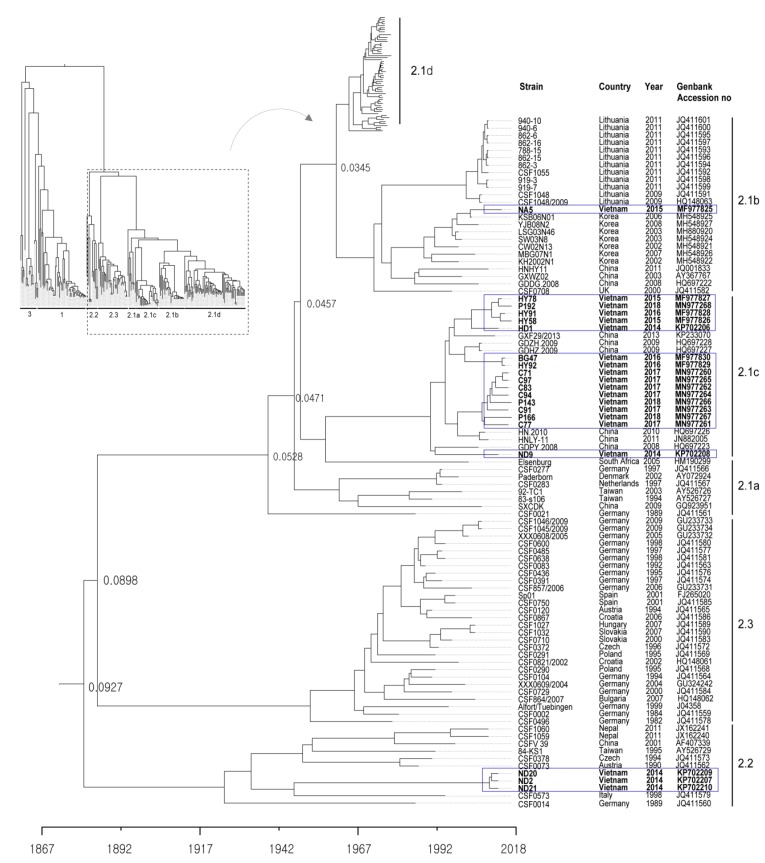
Phylogenetic tree based on the complete E2 sequences of Vietnamese CSFVs. The 171 complete E2 gene sequences, including those of 20 Vietnam strains, were obtained from the NCBI Genbank database. Each dataset was simulated using the following options: generation = 80,000,000; burn-in, 10%; and ESSs > 200. The confidence of the phylogentic analysis is represented by the numbers above the nodes representing branch length (time) based time scale by factor (1.0). Twenty Vietnamese strains are marked by blue boxes.

The 20 CSFVs identified from samples collected from the 16 backyard pig farms in north Vietnam between 2014 to 2018 were categorized as group 2.1b (one farm), 2.1c (14 farms), and 2.2 (one farms) based on nucleotide sequence analysis of the complete E2 gene (Table 1 and Figure 1 and Figure 2). Three strains (ND2, ND20 and ND21) belonged to sub-genotype 2.2, and an NA5 strain was classified as sub-genotype 2.1b; the 16 remaining strains were sub-genotype 2.1c (Table 1 and Figure 2). The mean time of the most recent common ancestor (tMRCA) for the Vietnamese strains was estimated to be 1932 (95% highest posterior density (HPD): lower, 1917; upper, 1957), with an effective sample size (ESS) of 729.1967 on the maximum clade credibility (MCC) tree. The clock rate (×10^−4^ substitutions/site/year) for Vietnamese strains was 8.49 (95% HPD: lower, 6.4415; upper, 10.589) (Figure 2). The phylogenetic tree suggested that strains belonging to sub-genotype 2.2 circulating in Vietnam are more closely related to strains isolated in European countries (Germany, Austria, the Czech Republic, and Italy) than to strains isolated in Asia (Taiwan, Nepal, and China) (Figure 2). The NA5 strain was on the sub-genotype 2.1b branch of the MCC tree and clustered with seven very similar strains (KSB06N01, YJB08B2, LSG03N46, SW03N8, CW02N13, MBG07N01, and KH2002N1) detected in Korea between 2002 and 2008 (Figure 2). The 16 Vietnamese CSFV strains (sub-genotype 2.1c) were most similar to CSFV strains GXF29/2013, GDZH.2009, GDHZ.2009, HN.2010, HNLY-11, and GDPY.2008 isolated in the southern regions (Guangdong, Guangxi, and Hunan) of China; phylogenetic analysis placed them in the same cluster (Figure 2). The complete E2 gene sequence of Vietnamese CSFV identified herein was submitted to GenBank under accession numbers KP702206–KP702210, MF977825-MF977830, and MN977260-MN977268. 

### 2.2. Comparison of the Pathogenicity of Different Sub-Genotypes

**Figure 3:**
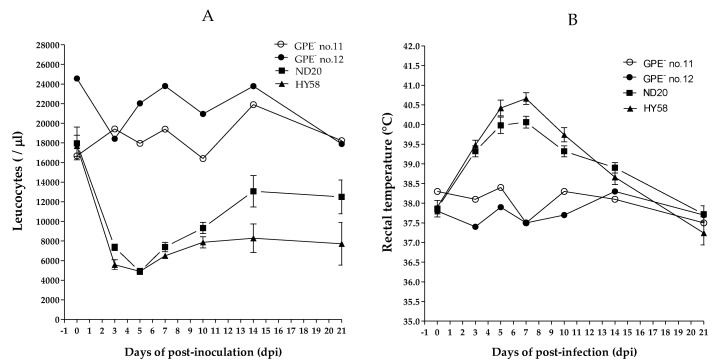
Leukocyte counts and rectal temperature of pigs inoculated with CSFV. Vietnamese strains inoculated group showed leukopenia and a high temperature. GPE^−^ vaccine control group was shown individually and other Vietnam CSFV groups shown as mean standard ± deviation. White blood cell count (**A**) and rectal temperature (**B**) were measured for 21 days post-infection.

Four of the five pigs in group 2, which were inoculated with the ND20 strain, survived for 30 days post-infection (dpi) (mortality, 20%); however, the mortality rate in pigs inoculated with the HY58 strain (group 3) was 60% (Table 2). The main clinical signs in pigs inoculated with the ND20 strain were fever, anorexia, and trembling; minor clinical signs included congestion, diarrhea, dehydration, and conjunctivitis. In addition to the clinical signs previously mentioned, pigs inoculated with the HY58 strain showed dehydration and hind leg paralysis (Table 2). The total mean clinical score at 21 dpi for pigs inoculated with ND20 and HY58 strains was 8.4 and 12.4, respectively (Table 3). According to the strain clinical sign scoring system, two Vietnamese strains (sub-genotypes 2.2 and 2.1c) were classified as having moderate virulence. At 7 days post-infection (dpi), the CSFV RNA copy number in the blood of pigs inoculated with the ND20 strain was lower (4.5–5.6 log_10_) than that of pigs inoculated with the HY58 strain (5.6–6.8 log_10_) (Table 2). At 0 to 21 dpi, leukocyte counts in pigs inoculated with the GPE^−^ strain (control group) were stable (average 18673/ul and 22840/ul, respectively). Pigs inoculated with the ND20 strain showed leukopenia, with leucocyte counts of 9000/ul from 3 to 7 dpi, whereas pigs inoculated with the HY58 strain had leukopenia from 3 to 21 dpi (Figure 3A). Both ND20 and HY58 strain infected pigs showed high rectal temperature (>40 °C) at 5–7 dpi (Figure 3B). 

### 2.3. Tissue Lesion Finding between Sub-Genotype 2.2 and 2.1c

**Figure 4:**
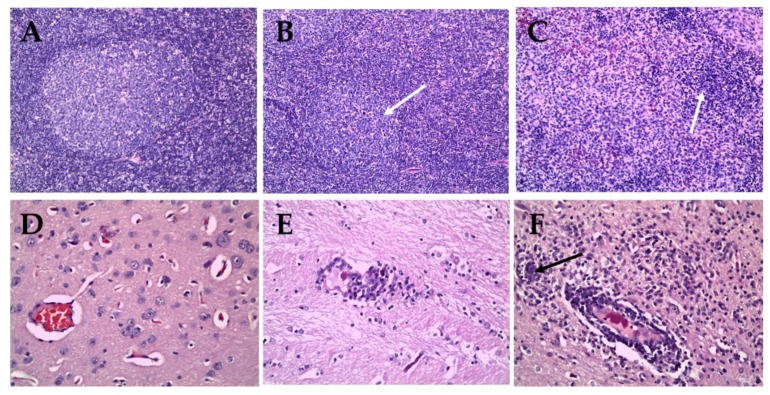
Histopathologic lesions in organs of pigs infected with GPE^−^ strain (**A**,**D**), Vietnamese CSFV ND20 strain (**B**,**E**) and HY58 strain (**C**,**F**). Nearly normal (A), mild (B, arrow), and severe (C, arrow) atrophy of follicle in lymph nodes of pigs (H&E stain; magnification × 200). Note focal hemorrhage and infiltration of macrophages (C). Normal brain parenchyma (D), mild perivascular cuffing (E), and severe PVC and glial nodule (F, arrow) in the brains of pigs (H&E stain; magnification × 400).

Pigs inoculated with the CSFV ND20 strain (sub-genotype 2.2) showed histopathologic lesions in various organs. Multifocal hemorrhage or nonspecific mild bronchopneumonia was observed in the lungs. Mild to moderate infiltration of macrophages and reticular cells around lymphoid follicles and lymphoid depletion in follicles were observed in the tonsils. Dilated tonsillar crypts contained cellular debris, keratin, and neutrophils. In addition, the proliferation of reticular cells and macrophages and atrophy of lymphoid follicles were confirmed in the lymph nodes (Figure 4B). The liver showed signs of multifocal pericholangitis, characterized by mild-to-moderate infiltration in the portal triad by lymphocytes and macrophages. Mild chronic interstitial nephritis and multifocal infiltration by lymphocytes and plasma cells was observed in the kidney. Perivascular cuffing (PVC), characterized by mild-to-moderate infiltration of the perivascular space (Virchow Robin space), was observed in the brain parenchyma, arachnoid space, and glial nodules (Figure 4E). However, there were no specific CSFV-associated lesions in the heart, spleen, ileum, or urinary bladder. CSFV HY58 strain (sub-genotype 2.1c) inoculated pigs showed more pathologic lesions in various organs compared to ND 20 group. The tonsils showed moderate infiltration of reticular cells and macrophages around lymphoid follicles, and severe lymphoid depletion in the follicles. In addition, cystic-dilated tonsillar crypts were also plugged with degenerated cellular debris, inflammatory cells, and keratin. Typical peripheral hemorrhage, proliferation of macrophages, and severe lymphoid depletion were observed in the lymph nodes (Figure 4C). Atrophy of the splenic white pulp and histiocytic infiltration were observed, along with non-suppurative interstitial nephritis and pericholangitis in the kidney and liver. Moderate-to-severe lesions typical of viral non-suppurative encephalitis, characterized by severe PVC, formation of glial nodules, and neuronophagia, were observed throughout the brain parenchyma (Figure 4F). However, no specific lesions were observed in the heart, ileum, or urinary bladder. There were no pathognomonic lesions or very mild histopathologic changes in the internal organs from pigs inoculated with the GPE^−^ strain (Figure 4A,D). Occasionally very mild atrophy of white pulp in spleen and mild interstitial nephritis were detected from the GPE^−^ strain inoculated group. 

### 2.4. Immunohistochemical Staining for Sub-Genotypes 2.2 and 2.1c

**Figure 5:**
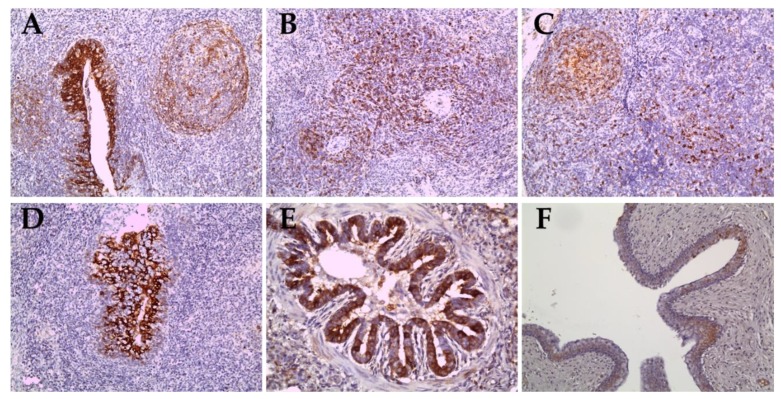
Immunohistochemical results in organs of pigs infected with the Vietnamese CSFV ND20 strain (**A**–**C**) and the HY58 strain (**D**–**F**). CSFV antigens were observed in the cryptal epithelium of the tonsils (A), infiltrated lymphocytes or macrophages of the spleen (B) and lymph nodes (C) of pigs with the ND20 strain (IHC; magnification × 200). Note CSFV antigens in the epithelial cells in the crypts of tonsil (D, IHC; × 200), the bronchiolar epithelium of the lung (E, IHC; × 400), and transitional epithelium of the urinary bladder (F, IHC; × 200) of pigs with the HY58 strain.

**Figure 6:**
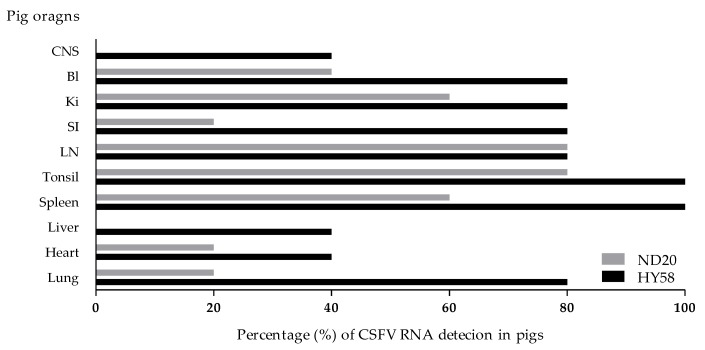
Ratio of CSFV-positive antigen detection in organs. CSFV RNA detection percentage from each organs of pigs (n = 5) infected with the Vietnamese CSF ND20 strain (light bar) and HY58 strain (black bar) by the qRT-PCR. CNS: central nervous system; BI: bladder; Ki: kidney; SI: small intestine; LN: lymph node.

In pigs inoculated with the ND20 strain (sub-genotype 2.2), immunohistochemical (IHC) analysis revealed specific CSFV antigens in 6 organs (lung, heart, spleen, tonsil, lymph nodes, and bladder) from animal (no. 16) dead at 28 dpi and in 3 organs (lymph nodes and ileum) from animal (no. 34) survived at 30 dpi (Table 2 and Table 4). However, most CSFV antigens were detected in lymphoid tissues such as tonsil, lymph node, and spleen (Figure 5A–C). In addition, CSFV antigens by the IHC analysis of pigs inoculated with the HY58 strain (sub-genotype 2.1c) were detected in all organs including CNS from animal (no. 29) dead at 23 dpi and in 5 organs (lung, tonsil, lymph nodes, ileum, and bladder) from animal (no. 31) survived at 30 dpi (Table 2 and Table 4 and Figure 5D–F). CSFV antigens were demonstrated not only in lymphoid tissues but also in other parenchyma including liver, urinary bladder, and brain. Histopathologically, CSFV antigens of pigs (no. 29 and no. 31) were observed in the cytoplasm of bronchial and bronchiolar epithelial cells in the lungs (Table 2 and Figure 5E), but focal deposits were observed in cardiac muscle cells in the heart of pig (no. 29) infected with the HY58 strain (Table 2 and Table 4). In the brain of pig (no. 29) inoculated with the HY58 strain, viral antigens were observed within infiltrating lymphocytes in PVC lesions (Table 2 and Table 3). QRT-PCR analysis of various organs from pigs inoculated with the ND20 strain detected a CSFV RNA copy number of 2.5 to 6.4 log _10_ (Table 4). CSF RNA copy number in all organs of pigs inoculated with the HY58 strain was higher (2.1–6.6 log _10_) than that in pigs infected with the ND20 strain (Table 4). The mean percentage ± SEM for CSF-positive organs of pigs infected with the ND20 strain and HY58 strain was 38.00% ± 9.638% and 72.00% ± 7.424%, respectively, the difference between two means was 34.00% ± 12.17% (Figure 6). 

## 3. Discussion

The most common clinical signs of CSF worldwide over the last 30–40 years have changed from acute to subacute, chronic, or asymptomatic disease [10,11]. In general, highly virulent CSFV strains (e.g., ALD, Brescia/IVI, and Eystrup) have high rates of morbidity and mortality when infecting domestic pigs and wild boar, regardless of age [12]. However, CSFV strains with moderate-to-low virulence may cause low or moderate morbidity and low mortality, and the rates can vary according to age, weight, and breed. Highly virulent CSFV strains spread rapidly throughout the body and are shed via all types of secretion [13]. By contrast, excretion of low virulent strains occurs only via the oronasal route because these strains are restricted to specific target organs [13]. Previous studies suggest that this difference in secretion pattern between highly virulent strains and low virulent strains is due to virus tropism [3,13]. Genetic analysis of recent genotypes 2 and 3 suggests that they are less virulent than the old genotype 1. A phylodynamic study suggests that genotype 2 has emerged via an antigenic avoidance phenomenon because live attenuated CSF vaccines were based on genotype 1 [14]. Overall, we found that Vietnamese CSFV genotype 2 had moderate pathogenicity; however, there were some pathogenic differences between sub-genotypes 2.2 and 2.1c. Total mean clinical score of sub-genotype 2.1c (HY58 strain) was slightly higher than sub-genotype 2.2 (ND20 strain), even though they are all in the range of the “moderate” score. The overall histopathologic lesions (including lymph node and brain) associated with CSFV in this study were more frequent and severe in pigs inoculated with the HY58 strain than pigs inoculated with the ND20 strain. The antigenic distributions by the IHC staining analysis, correlated with histopathologic lesions, were also more wide and intense in the internal organs (lymphoid tissues, liver, urinary bladder, and brain) with the HY58 strain than those with the ND20 strain. The HY58 strain belonging to sub-genotype 2.1c caused higher mortality, caused more lesions in organs/tissues, had a higher CSFV RNA copy number, induced higher rectal temperatures, and caused more severe leukopenia than the ND20 strain (sub-genotype 2.2). Although the HY58 strain caused 60% mortality in 30-day-old pigs within 30 days after inoculation, we expect it to be less pathogenic in heavier and/or older pigs. Comparative genetic analysis between the ND20 (sub-genotype 2.2) and HY58 (sub-genotype 2.1c) strains isolated from Vietnam revealed sequence identities of 86.7% for the N^pro^ gene, 87.9% for the E^rns^ gene, 88.3% for the E1 gene, 86.1% for the E2 gene, 88.9% for the NS4B gene, and 86.1% for the NS5A gene [15]. The NA5 strain detected in the Nghe An region in north Vietnam in 2015 belonged to sub-genotype 2.1b, as did CSFV strains circulating among Korean domestic pigs from 2002 to 2009 [16]. The NA5 strain showed high sequence similarity with seven Korean CSFV strains (KSB06N01, YJB08B2, LSG03N46, SW03N8, CW02N13, MBG07N01, and KH2002N1): 96.7–99.5% at the nucleotide level and 97.3–99.5% at the amino acid level. The Korean sub-genotype 2.1b strains showed moderate pathogenicity when inoculated into pigs and did not show high pathogenicity, even in field CSF outbreaks [16,17]. One of two landrace pigs infected with strain SW03 (10^6.0^TCID_50_/mL) died at 18 dpi, but one lived until 21 dpi [17]. The viral RNA copy number in organs/tissues from the two pigs infected with the SW03N8 strain was somewhat lower (10^1.08−4.82^ log_10_) in tonsil, lung, heart, mesenteric lymph node, and cecum than that with the YC11WB strain [17]. Therefore, we think that based on its genetic similarity with the Korean CSFV sub-genotype 2.1b, the Vietnamese NA5 strain has moderate virulence. Genotype 2 is the prevalent genotype in countries (Cambodia, Laos, and Thailand) around Vietnam [7,8,9]. Interestingly, CSFV strains detected in the Guangdong, Guangxi, and Hunan regions in southern China from 2008 to 2013 are closely related to Vietnamese CSFV genotype 2.1c; indeed, genetic analysis revealed that they belonged to the same cluster. Therefore, it may be that direct and indirect transmission of CSFV occurred via livestock movements or livestock vehicle movements between the two countries. The phylogenetic tree suggests that sub-genotype 2.2 strains circulating in Vietnam are more closely related to strains isolated from European countries (Germany, Austria, the Czech Republic, and Italy) than to strains isolated in Asia (Taiwan, Nepal, and China). Here, we show that the complete E2 nucleotide sequence of strain ND20 is more similar to that of strains isolated from European countries (CSF0378, CSF0573, CSF0073, and CSF0014) (96.3–97.6%) than to that of strain 84-KS1 (96.1%) isolated from Taiwan [18], or to that of strain CSF1059 (94.1%) isolated from Nepal [19]. The finding that the Vietnamese strains within subgroup 2.2 are similar to strains isolated in Europe is interesting because it is unclear how they entered Vietnam; further genetic analyses of strains from neighboring countries (e.g., Cambodia, Laos, and China) may clarify this. 

In conclusion, sub-genotype 2.1c is the major genotype of CSFV in northern Vietnam, which is a geographically important location. Sub-genotype 2.1c, which is prevalent in northern Vietnam, has characteristics similar to those of strains isolated in the Guangdong, Guangxi, and Hunan regions in Southern China, suggesting that mutual exchange between the two regions will continue. The pathogenicity of genotype 2 in Vietnam is moderate, although that of sub-genotypes 2.1c is slightly higher than that of sub-genotype 2.2. Further genetic and pathogenic analyses of Vietnamese CSFV strains will help us to understand their characteristics, thereby enabling improved control strategies and policies for control of CSF in Vietnam. 

## 4. Materials and Methods

### 4.1. Samples, RT-PCR, Phylogenetic Tree, and Virus Isolation

Blood samples from pigs (30–90 days old) with suspected CSF were collected from 16 pig farms from June 2014 to December 2018. Samples were collected from farms in Xuan Truong-Nam Dinh, Hi Duong, Nghe An, Hung Yen, Bac Giang, My Xa-Nam Dinh, and Ninh Giang-Hai Duong (Table 1 and Figure 1). None of the pigs on the 16 pig farms were vaccinated. Total RNA was extracted from blood using a micro column-based QIAamp Viral RNA Mini kit (Qiagen, USA) to identify CSFV. The RT-PCR conditions and specific primers used to amplify the complete E2 gene have been reported previously [4]. The complete E2 gene sequences of CSFVs (including the Vietnam strains examined in this study) were obtained from the NCBI GenBank database and aligned using the CLUSTAL X alignment program. Next, a BEAST input file was generated using BEAUti within BEAST package v1.8.1 [20]. Rates of nucleotide substitution per site per year and the tMRCA were estimated using a Bayesian MCMC approach. The exponential clock and expansion growth population model in the BEAST program was used to obtain the best-fit evolutionary model and the MCC tree was visualized using Figtree 1.4 [21]. For virus isolation, CSFV-positive blood samples were inoculated to porcine kidney 15 (PK-15) cell (grown to 80% confluence in 6-well plates) using alpha-minimum essential medium (GIBCO Cat. No. 12571-063) with L-glutamine (GIBCO Cat. No. 25030-081), sodium pyruvate (GIBCO Cat. No. 11360-070), and antibiotic-antimycotic solution (GIBCO Cat. No. 15240-062). The six-well plate (PK-15 cells) were inoculated for 3–5 days at 37 °C and the virus was identified by immunochemical staining using the CSFV monoclonal antibody 3B6 (Median Diagnostics Co., South Korea). 

### 4.2. Animal Experiments 

After virus inoculation, pigs were monitored to confirm the pathogenicity of two isolated sub-genotypes (2.2 and 2.1c). ND20 strain (sub-genotype 2.2) and HY58 strain (sub-genotype 2.1c) were each inoculated into pigs (30 days old) via two simultaneous administration (2 mL orally and 2 mL intramuscularly) (10^5.0^ TCID_50_/mL). GPE^−^ strain, a CSFV vaccine strain used in Japan, was inoculated via the same route and dose as a control. Pigs were divided into group 1 (n = 2, GPE^−^ strain), group 2 (n = 5, ND20 strain), and group 3 (n = 5, HY58 strain), and observed for 30 days. To detect CSFV RNA copies and to check for leukopenia (leucocyte counts below 9000/μL), blood samples were collected at 0, 3, 5, 7, 10, 14, and 21 dpi. During the course of the experiment, pigs were monitored daily for clinical signs (anorexia, diarrhea, dehydration, tremble, congestion, conjunctivitis, and hind leg paralysis). The clinical score was determined by ten parameters following previous procedure [22]. Briefly, ten parameters (liveliness, body tension, body shape, breathing, walking, skin, eyes, appetite, defecation, and leftovers in feeding trough) were graded according to the following scoring system: 0, normal; 1, slightly altered; 2, showing distinct clinical signs; and 3, showing severe CSF clinical signs. Virus strains were classified as highly virulent (total clinical score: >15), moderately virulent (5–15), low virulent (<5), or avirulent (0) [22]. Rectal temperature was checked at the time of blood collection. Pigs were necropsied and ten organs (lung, heart, liver, spleen, tonsil, lymph node, intestinal, kidney, bladder, and central nervous system) were examined to detect histopathologic lesions and the presence of CSFV antigens. Collected tissues were processed routinely for histopathologic examination and stained with hematoxylin and eosin (H&E). Mortality was calculated over the 30-day observation period. 

### 4.3. Immunohistochemical Assay and qRT-PCR 

IHC staining was performed as described previously [23,24]. CSF antigens derived from the ND20, HY58, and GPE^−^ strains were detected using DAB and the EnVision^TM^ peroxidase-conjugated polymer reagent (DAKO, Denmark). The results were scored according to the strength of straining as follows: +: 1–3 foci/section, ++: 4–10 foci/section, +++: >10 foci/section. The CSF RNA copy number within the organs of pigs was measured using quantitative real-time PCR (qRT-PCR) and calculated as log 10/g. The AnyQ CSFV qRT-PCR (Median Diagnostic Co. Cat No. NS-CSF-31, Korea) system and TaqMan probes targeting the 5’-UTR region with high specificity were used. The reaction conditions for qRT-PCR have been reported previously [24]. IHC staining was performed on the pigs showing most severe or mild clinical signs of each group. Whereas, qRT-PCR was performed on all pigs of each group.

## Figures and Tables

**Table 1:** Vietnamese strains of classical swine fever virus examined during the 5-year study.

Date of Collection	Place	Farm Name	Age of Sampled Pig	Strain	CSFV Genotype	Accession Number
June, 2014	^a^ Nam Dinh	XTND	60	ND2	2.2	KP702207
June, 2014	^a^ Nam Dinh	XTND	60	ND20	2.2	KP702209
June, 2014	^a^ Nam Dinh	XTND	60	ND21	2.2	KP702210
June, 2014	^b^ Nam Dinh	NDB	90	ND9	2.1c	KP702208
July, 2014	^c^ Hai Duong	NGHD	30	HD1	2.1c	KP702206
Jan, 2015	Nghe An	NAN	30	NA5	2.1b	MF977825
June, 2015	Hung Yen	HYen	70	HY58	2.1c	MF977826
Sep, 2015	Hung Yen	HYen	70	HY78	2.1c	MF977827
Feb, 2016	Hung Yen	HYN	50	HY91	2.1c	MF977828
Mar, 2016	Hung Yen	HYY	60	HY92	2.1c	MF977829
Mar, 2016	Bac Giang	BGiang	61	BG47	2.1c	MF977830
Jul, 2017	Hung Yen	HYenI	30	C71	2.1c	MN977260
Aug, 2017	Hung Yen	HYenJ	30	C77	2.1c	MN977261
Aug, 2017	Hung Yen	HYenK	40	C83	2.1c	MN977262
Sep, 2017	Hung Yen	HYenJ	30	C91	2.1c	MN977263
Sep, 2017	Hung Yen	HYenL	60	C94	2.1c	MN977264
Sep, 2017	Hung Yen	HYenM	50	C97	2.1c	MN977265
Oct, 2018	Hung Yen	HYenN	30	P143	2.1c	MN977266
Nov, 2018	Nam Dinh	NDinhO	40	P166	2.1c	MN977267
Dec, 2018	Ha Nam	Ha NamP	40	P192	2.1c	MN977268

^a^ Nam Dinh: Xuan Truong-Nam Dinh; ^b^ Nam Dinh: My Xa-Nam Dinh; ^c^ Hai Duong: Ninh Giang-Hai Duong.

**Table 2:** CSFV RNA copy number in blood and clinical signs in pigs infected with CSFV.

Group	Pig No.	SG	^a^ Strain	OP	DOP	^b^ Clinical Signs	CSFV RNA Copy Number (log _10_) in Blood (Days of Post-Infection)						
							0	3	5	7	10	14	21
1	11	1.1	GPE^−^	30	alive	None	-	-	-	-	-	-	-
	12	1.1	GPE^−^	30	alive	None	-	-	-	-	-	-	-
2	7	2.2	ND20	30	alive	F, A, Di	-	3.6	5.3	5.6	3.2	3.5	2.7
	8	2.2	ND20	30	alive	F, A, T, Co	-	2.7	4.1	5.3	3.8	3.1	2.4
	9	2.2	ND20	30	alive	F, A, T, Di	-	3.0	5.5	5.1	3.3	4.2	1.8
	16	2.2	ND20	30	28	F, A, Di, T, C	-	3.4	5.8	5.4	4.5	2.5	2.9
	34	2.2	ND20	30	alive	F, A, T, C	-	2.5	4.9	4.5	2.7	2.6	1.7
3	21	2.1c	HY58	30	alive	F, A, De, T	-	3.2	5.7	5.9	5.2	3.5	3.7
	24	2.1c	HY58	30	22	F, A, Di, De, C, T	-	2.7	6.4	6.1	4.3	4.2	3.5
	29	2.1c	HY58	30	23	F, A, De, C, HP	-	2.9	6.1	6.8	5.7	5.3	3.7
	31	2.1c	HY58	30	alive	F, A, De, T, Co	-	3.3	4.7	5.6	5.8	4.2	2.6
	38	2.1c	HY58	30	25	F, A, Di, T, Co, HP	-	3.6	5.4	6.7	4.9	4.8	3.5

^a^ Strain: an inoculation of virus (4 × 10^5.0^TCID_50_/mL/dose) was administered via the oral and intramuscular routes (half of the dose via each of these routes). SG: sub-genotype; OP: observation period (days); DOP: day of death during observation period. ^b^ Clinical signs were F (fever), A (anorexia), Di (diarrhea), De (dehydration), T (tremble), C (congestion), Co (conjunctivitis), and HP (hind leg paralysis).

**Table 3:** Clinical score of pigs infected with CSFV.

	Mean Clinical Sore (21 dpi)										Total Mean Clinical Score	Virulent
Parameter	*Li	BT	BS	Br	Wa	Sk	Ey	Ap	De	Le		
ND20 strain	0.8	0.2	0.5	0.8	0.8	0.9	1.0	1.5	0.5	1.4	8.4	moderate
HY58 strain	1.4	1.0	1.2	1.2	1.2	1.0	1.0	1.6	0.8	2.0	12.4	moderate

*Li: liveliness; BT: body tension; BS: body shape; Br: breathing; Wa: walking; Sk: skin; Ey: eyes; Ap: appetite; De: defecation; Le: leftovers in feeding trough.

**Table 4:** The qRT-PCR and immunohistochemical staining results for various pig organs.

*G	Strain	Pig No.	CSF RNA Copy Number (log 10)/Foci Score									
			Lung	Heart	Liver	Spleen	Tonsil	LN	SI	Ki	Bl	CNS
1	GPE^−^	11	-	-	-	-	-	-	-	-	-	-
		12	-	-	-	-	-	-	-	-	-	-
2	ND20	7	-	-	-	3.3/*	4.5/*	-	-	4.3/*	3.1/*	-
		8	-	-	-	-	3.7/*	3.8/*	-	2.5/*	-	-
		9	-	-	-	4.1/*	4.9/*	3.1/*	-	-	-	-
		16	3.3/+	4.1/+	-	5.2/+	6.4/+++	6.2/++	-	3.7/-	3.8/+	-
		34	-	-	-	-	3.6/-	5.4/+	4.5/+	-	-	-
3	HY58	21	-	2.1/*	-	5.2/*	5.7/*	4.2/*	3.4/*	-	-	-
		24	4.4/*	-	-	4.1/*	5.9/*	4.6/*	-	3.8/*	4.8/*	-
		29	5.1/++	4.2/+	4.6/+	5.5/+	6.6/++	6.1/++	5.3/++	3.5/+	5.2/++	5.8/++
		31	4.7/+	-	-	3.7/-	6.2/++	5.9/+	4.8+	3.6/-	4.7/+	-
		38	3.5/*	-	3.4/*	4.8/*	5.2/*	-	3.9/*	4.6/*	3.1/*	4.5/*

*G: group. +: 1–3 foci/section, ++: 4–10 foci/section, +++: >10 foci/section. LN: lymph node; SI: small intestine; Ki: kidney; Bl: bladder; CNS: central nervous system. *: not tested.
